# Structure Inheritance in Nanoparticle Ink Direct-Writing Processes and Crack-Free Nano-Copper Interconnects Printed by a Single-Run Approach

**DOI:** 10.3390/ma12091559

**Published:** 2019-05-13

**Authors:** Shujie Liu, Yujie Li, Songling Xing, Lei Liu, Guisheng Zou, Peng Zhang

**Affiliations:** 1 School of Materials Science and Engineering, Harbin Institute of Technology at Weihai, Wenhua West Road 2, Weihai 264209, China; liushujie_lsj@163.com; 2 Department of Mechanical Engineering, Harbin University of Science and Technology at Rongcheng, Xueyuan Road 2006, Rongcheng 264300, China; 3 Department of Mechanical Engineering, State Key Laboratory of Tribology, Key Laboratory for Advanced Manufacturing by Materials Processing Technology, Ministry of Education of PR China, Tsinghua University, Beijing 100084, China; xingsl14@mails.tsinghua.edu.cn (S.X.); liulei@tsinghua.edu.cn (L.L.); zougsh@tsinghua.edu.cn (G.Z.)

**Keywords:** nano-copper conductive ink, interconnects, structure inheritance, crack-free, direct-writing

## Abstract

When nanoparticle conductive ink is used for printing interconnects, cracks and pores are common defects that deteriorate the electrical conductivity of the printed circuits. Influences of the ink solvent, the solid fraction of the ink, the pre-printing treatment and the sintering parameters on the interconnect morphology and conductivity were investigated. It was found that the impacts of all these factors coupled with each other throughout the whole procedure, from the pre-printing to the post-printing processes, and led to a structure inheritance effect. An optimum process route was developed for producing crack-free interconnects by a single-run direct-writing approach using home-made nano-copper ink. A weak gel was promoted in the ink before printing in the presence of long-chain polymers and bridging molecules by mechanical agitation. The fully developed gel network prevented the phase separation during ink extrusion and crack formations during drying. With the reducing agents in the ink and slow evaporation of the ink solvent, compact packing and neck joining of copper nanoparticles were obtained after a two-step sintering process. The crack-free interconnects successfully produced have a surface roughness smaller than 1.5 μm and the square resistances as low as 0.01 Ω/□.

## 1. Introduction

Conductive ink composed of metal nanoparticles has been successfully used for printing interconnects and other functional parts for various electronic devices, such as electronic contact lenses [1], antennas [2,3], flexible displays [4,5], and chemical probes [6]. Simple direct-writing techniques are preferred. However, the printed structures are frequently subject to pores [7] and cracks [8,9,10]. In some cases, the cracks can be applied as sensors to percept external loads [11] and the pores as detectors for molecules like glucose [12]. But for the interconnect structures, cracks and pores are defects that destroy their continuity and compactness and the electrical conductivity is therefore deteriorated. Moreover, fractures and breaks are easily developed in the interconnects with dense cracks and large pores. Complete failure of the corresponding electrical devices and systems then takes place. 

In order to solve these problems, multiple printing or multi-layer coating methods with up to 10 or more steps [8] are used to obtain crack-free nanoparticle films at the expense of alignment accuracy and productive efficiency. Post-printing treatment like sintering is also used to improve the microstructure and electrical properties of the printed interconnects. High sintering temperature can smooth the porous mesh and effectively lower the resistivity [13] but may also do harm to the substrate materials and the electrical devices. Sintering made in ovens under pressure [14] and laser sintering [15,16] both increase the compactness of the printed structures. With such techniques, expensive equipment and complex process control are necessary and the total cost rises up. To produce compact, crack-free interconnects by a single-run printing process with simple post-treatment techniques is still a big challenge. 

Studies on the formation mechanisms of the cracks and pores help a lot in controlling these defects. Thiery [17] and Yang [18] believed that the cracks appeared in the drying process. The crack density depended on the initial solid fraction of the ink gel as well as the adhesion of the printed material on the substrate surface. During solvent evaporation, the nanoparticles in the printed structures were rearranged, causing the layer spacing to collapse and the following disintegration to form cracks and large pores. Dugyala [19] studied the width of the cracks and concluded that it was determined by the initial concentration of the nanoparticles and their shapes. Yet Yang et al [20] stated that the cracks originated from the release of stresses, which was caused by the solvent loss and the thermal expansion of the printed structure itself. 

Actually, the cracks and pores may form at any stage during the printing and post-treatment processes. Original properties of the nanoparticle conductive ink, the printing process control and the post-printing treatment all have great influences on the final macro- and micro-structures of the printed interconnects. Furthermore, the pre-printing, the printing, and the post-printing processes may have complex coupling effects with each other. To control just a single one or a few of them is far from enough in eliminating the cracks and pores. In this work, the nano-copper particles were made into conductive inks and used for direct-writing of the interconnect structures. The printing procedure was studied as an integrated process rather than a simple combination of separate steps. Effects of the solvent, the solid fraction of the ink, the pre-printing treatment and the post-printing sintering parameters on the interconnect morphology and conductivity were investigated. Special attention was focused on the coupling interactions of all these factors during the whole pre-printing, printing and post-printing processes. Structures formed in the ink before it was touching the substrate surface was found to have obvious influences on the final structures of the interconnects. A structure inheritance throughout the overall printing process was found. Accordingly, an optimum processing route was proposed to obtain compact, crack-free interconnects with low surface roughness and high conductivity by a single-run direct-writing approach. A weak gelation was promoted in the ink through a mechanical-ultrasonic combined pretreatment. This helped in solving problems like phase separation of the ink during extrusion and crack formations during drying. Sintering simply conducted in an oven at 250 °C was sufficient for obtaining interconnect structures with the square resistance as low as 0.01 Ω/□.

## 2. Materials and Methods 

### 2.1. Preparation of the Nano-Copper Conductive Ink

Sodiumdodecylsulphate (SDS), poly(vinylpyrrolidone) (PVP-K30), NaOH, CuSO_4_, Ethylene glycol, ethanol, and hydrazine hydrate (80 wt % of hydrazine monohydrate) were all of analytical grade and used as purchased. Deionized water produced by a Molecular Molelement 1805A system had a resistivity of 18.25 MΩ·cm. Copper nanoparticles were synthesized by a double template method given in Reference [21]. Briefly, 9 mmol/L SDS, 6 g/L PVP, and 30 mmol/L CuSO_4_ were added in 150 mL water successively and stirred at 1800 rpm. The pH value of the solution was then adjusted to be about 10 by dropping in saturated NaOH aqueous solution. After 24.3 mL hydrazine hydrate was mixed in, the solution was heated to 50 °C. And then hydrazine hydrate was added for the second time, with 14.6 mL in volume. The final solution was kept at 50 °C and treated ultrasonically for 30 min. 

The copper nanoparticles obtained were first separated from the final reaction solution by centrifuging at 12,000 rpm for 3 min and then dispersed again in water to dissolve extra organics and reactants. Finally, the nanoparticle powder was separated out. Organics remained in the powder were measured to be less than 2% in our previous work (Reference [22]). The nanoparticles were added to various solvents to produce conductive inks. The nano-copper inks with solid fractions of 20, 30, 40, and 50 wt % were prepared and used. The inks were ultrasonically treated for 5 min to assure uniform dispersion of the particles. 

### 2.2. Printing and Sintering of the Nano-Copper Interconnects

**Figure 1:**
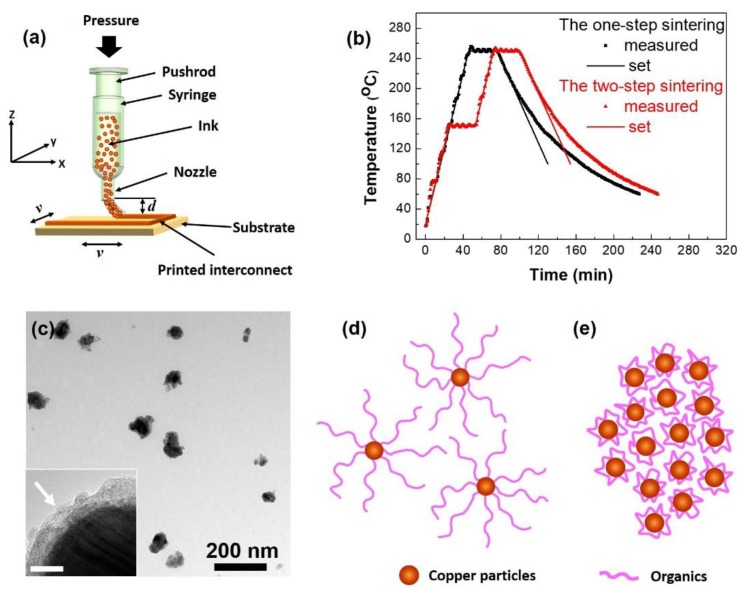
(**a**) A schematic picture of the home-made direct-writing installation. (**b**) The temperature profiles set and measured for both the one-step and two-step sintering strategies. (**c**) A typical TEM image of the nano-copper particles. The inset in (**c**) is a high resolution TEM image of a particle. The organic layer capped around the particle is pointed out by a white arrow. The scale bar in the inset stands for 5 nm. (**d**) Particles dispersed in the ink solvent by the steric hindrance effect as the organic chains extended fully and (**e**) particles aggregated when the organic chains curled up.

Nano-copper interconnect structures were printed on substrate surfaces by a home-made direct writing installation as shown schematically in Figure 1a. Glass substrates were used. The nano-copper ink was loaded in a syringe with a nozzle of 0.7 mm in inner diameter. Pressure was applied at the top end of the pushrod and the conductive ink was extruded from the nozzle. When uniform extrusion of the ink was expected, a stepper motor was used to apply the pressure. The glass substrates were fixed on a sample platform, which was moving in the XY-plane at an expected velocity *v* by a set of stepper motors. The distance between the nozzle tip and the substrate surface *d* was 0.2–0.4 mm, adjusted according to the properties of the ink and the moving speed of the sample stage. All of the interconnect structures were printed by just a single-run printing process.

After being printed and dried in air, the nano-copper interconnect structures were sintered in an oven with N_2_ supplied as a protection. Two kinds of temperature rising strategies were used. One was a one-step heating process. The sample was heated directly from the room temperature to 250 °C at a heating rate of 5 °C/min. The other was a two-step heating process. The temperature was first risen to 150 °C at a heating rate of 5 °C/min and kept constant at this value for 30 min. And then the temperature was further increased to 250 °C, again at a heating rate of 5 °C/min. For both cases, after the sample temperature reached 250 °C, it was kept unchanged for 30 min. Sintering of the nano-copper interconnect structures were accomplished at this temperature. Finally, the samples were cooled down to the room temperature at a cooling rate of 2–3 °C/min. A thermocouple was placed at the sample position in the oven. The temperature profiles measured for both the one-step and two-step sintering strategies were plotted and compared with the set values in Figure 1b.

### 2.3. Characterization

A Tecnai G2 F30 field emission transmission electron microscope (FETEM, FEI, Hillsboro, USA) was used for recording images of the nanoparticles. The thickness and the surface roughness of the printed interconnect structure were determined by imaging the cross sections and the morphologies of the printed interconnects with an Olympus stereo microscope (OLYMPUS DSX510, Olympus Corporation, Tokyo, Japan). X-ray diffraction (XRD, Bruker D8, Billerica, USA) spectra were acquired with a Bruker D8 Advance X-ray diffractometer (Cu Kα). The square resistances of the sintered interconnect structures was measured by a four-probe meter (RST-8, Guangzhou, China). The microstructures of the nano-copper interconnect structures both before and after sintering were observed by a metallographic optical microscope (XYMRT, Sunny Optical Instruments, Yuyao, China) and a VEGAII SBH scanning electron microscope (SEM, TESCAN, Brno, Czech).

## 3. Results and Discussion

Nano-copper ink has attracted increasing interest due to the high electrical properties and low cost of copper. In our previous work [22], copper nanoparticles were synthesized and studied attentively. The nanoparticles synthesized by the double template method had diameters of 20–100 nm and capped by a layer of PVP-SDS on their surfaces as shown in Figure 1c. The extension of the long chains of the organic capping agents in a certain solvent could be controlled by the solubility of the organics, the pH value and the ionic strength of the solution [22]. In a neutral solution, the organic chains extended fully into fingerlike structures as shown in Figure 1d and the particles dispersed uniformly in the solvent due to the steric hindrance effect. While in an alkaline solution, the organic chains curled up due to flocculation [22]. The particles were then aggregated (Figure 1e).

Groups of experiments were made to study the influences of the solid fraction of the nanoparticle ink, the solvent properties, the sintering parameters, and the pre-printing treatment process on the crack formation and the electrical properties of printed structures. To avoid tedious details, only optimized results at the specified conditions were given.

### 3.1. Influence of the Solid Fraction of the Nano-Copper Ink on the Printed Structure Morphologies

**Figure 2:**
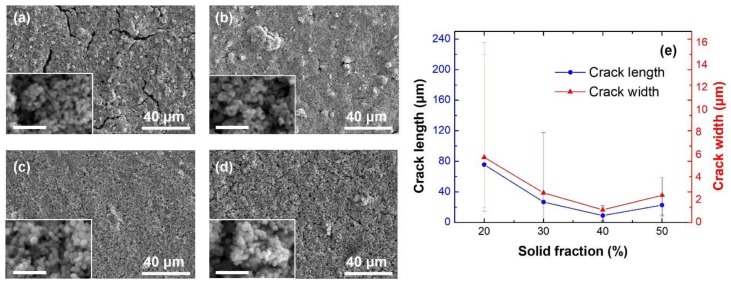
Interconnects prepared by using nano-copper inks with solid fractions of (**a**) 20%, (**b**) 30%, (**c**) 40% and (**d**) 50%, respectively. The insets show microstructures at large magnifications. The scale bars in the insets stand for 500 nm. (**e**) The length and width of cracks in the interconnects varying with the solid fractions of the inks. Water was used as the ink solvent.

The nano-copper ink used for preparing the structures in Figure 2 was prepared by adding nano-copper particles of 20, 30, 40, and 50 wt % in content, respectively, to water. The printing parameters, including the extrusion speed, the height of the nozzle tip and the moving speed of the sample platform for each ink specimen were optimized to assure that the printed structures had clear outlines, uniform thicknesses, and the least cracks. The microstructures of the printed interconnects were compared in Figure 2. The one-step sintering process was used to post-treat the interconnects. When the solid content of the ink was too low, for example 20 wt %, large cracks with lengths up to 100 μm appeared unavoidably (Figure 2a). At 30 wt % of the copper particles, extremely large cracks could be removed by optimizing the printing parameters. However, small cracks of 20–50 μm in length still existed, as shown in Figure 2b. The most compact interconnect structures were obtained at 40 wt % of the solid content in the ink (Figure 2c). Increasing the solid content further to 50 wt %, no large cracks were found but small cracks and large pores as shown in Figure 2d spread all over the sample. The length and width of cracks were measured from the SEM images and plotted in Figure 2e according to the solid fractions of inks. For each case, twenty specimens were used for statistics. The average values of the crack length and the crack width were denoted by the symbols in Figure 2e. The maximum and minimum values were used to define the error bars. The bulk lines were provided just to guide the eye. Rather similar data were found for solvents other than water and in the following sections 40% was used as a preferred value for the solid fraction of the ink.

### 3.2. Influence of the Ink Solvent on the Printed Structure Properties

**Figure 3:**
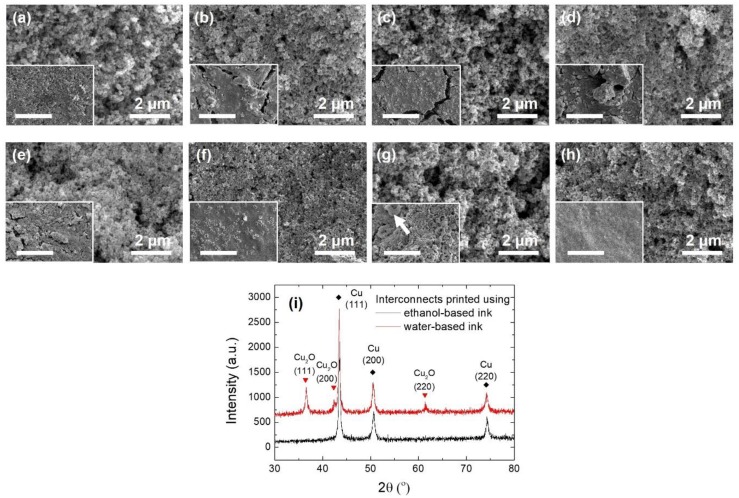
Interconnects prepared by using nano-copper inks with solvents of (**a**) water, (**b**) ethylene glycol, (**c**) ethanol, (**d**) the hydrazine hydrate solution, (**e**) the hydrazine hydrate solution mixed with ethylene glycol, (**f**) the hydrazine hydrate solution mixed with ethanol, (**g**) ethylene glycol mixed with ethanol and (**h**) a mixed solvent composed of the hydrazine hydrate solution, ethylene glycol and ethanol, respectively. The insets show microstructures at small magnifications. The scale bars in the insets stand for 40 μm. The white arrow in the inset of (**g**) marks out a block of organic material. (**i**) Typical XRD patterns measured for interconnects printed with the water- and ethanol-based inks. Quite a lot Cu_2_O was detected in the interconnect printed with the water-based ink.

With a fixed solid content of 40 wt %, eight different kinds of solvents, namely water, ethylene glycol, ethanol, an aqueous solution of hydrazine hydrate (6 vol %, indicated as the hydrazine hydrate solution in the following text), the hydrazine hydrate solution mixed with ethylene glycol (1:1 volume ratio), the hydrazine hydrate solution mixed with ethanol (1:1 volume ratio), ethylene glycol mixed with ethanol (1:1 volume ratio), and a mixed solvent composed of the hydrazine hydrate solution, ethylene glycol and ethanol (1:2:1 volume ratio), were used to prepare the inks, respectively. Typical microstructure images recorded from the interconnects printed with optimized printing parameters developed in each case and post-treated by the one-step sintering process were compared in Figure 3.

Among these solvents, water, ethylene glycol and ethanol were single solvents. With water, printed structures without cracks could be obtained by optimizing the printing parameters (Figure 3a) as already shown in Section 3.1. But their square resistances were quite large, about 3–5 × 10^5^ Ω/□. Large cracks were found for structures printed with the glycol- and ethanol-based inks (Figure 3b,c, respectively), while the electrical properties of the printed structures obtained in these two cases were much better. The square resistances measured were seven orders of magnitude lower (0.04 Ω/□). The differences in the printed interconnect morphologies were mainly due to the differences in the extending states of the capping organics around the nanoparticles in these solvents. The solubility, the chain extending states and the molecule configurations of PVP in different solutions were found to vary largely according to the polarity of the solvents [23]. The polarities of water, glycol, and ethanol decrease in turn. The outspreading of PVP chains, and thus the film formation ability of PVP, diminishes accordingly. In this work, the worst situation was with ethanol. Large and dense cracks appeared even before the printed structures became completely dry. After being sintered, dense pores were also detected. Rapid evaporation of ethanol aggravated the emergence of cracks and pores. The large differences in the electrical properties originated from the reducibility of the solvents. In water, nano-copper particles might be partially oxidized. While glycol and ethanol could protect them from being oxidized to some extent. Typical XRD patterns of the films printed with the water- and ethanol-based inks were compared in Figure 3i. Obviously, less oxides were detected in the case of the ethanol-based ink and this resulted in much lower square resistances in spite of the cracks.

When N_2_H_4_·H_2_O was added in water, strong reducibility of the solution was present. Oxidized nano-copper particles were reduced again and residual reactants remained from the synthesis process could also be reduced into nano-copper particles. The interconnect structures printed by the ink with the hydrazine hydrate solution had an obvious metallic luster. The square resistances were about 0.12 Ω/□. But according to our previous studies, rather high pH value of the ink solution could lead to flocculation of PVP [22]. The film formation ability of PVP was consequently deteriorated and the printed structures were found to have dense fissures, as shown in Figure 3d.

Mixing the hydrazine hydrate solution with glycol or ethanol, the anti-oxidation effect was revealed by clear metallic luster of the interconnect structures as well. However, the electrical properties became worse to varying degrees. This was again due to the degeneration of the film formation ability of PVP at high pH values. Except for the cracks, pores of diversified sizes existed, as shown in Figure 3e,f. Large defects were more likely to form when the component solvent of ethanol mixed with the hydrazine hydrate solution was used. Fracturing and porosifying were intensified by the large solvent loss rate. Pores as large as several micrometers were detected in this case and the square resistances of the printed interconnects were as high as 1.6 × 10^5^ Ω/□. 

When glycol and ethanol were mixed together, although problems caused by fast evaporation of ethanol could be partially resolved, dense fissures and large pores were found here and there in the microstructure images (Figure 3g). Blocks of organic materials as indicated in the inset of Figure 3g by a white arrow also presented locally. The reason was still not clear, although, it may be due to the segregation of PVP in mixed solutions [24,25]. The fissures, pores and blocks of organics were all bad for electrical properties of the interconnects and the square resistances were very large, about 3.6 × 10^6^ Ω/□.

Uniform microstructure of the prepared interconnects was gained when the mixed solvent composed of the hydrazine hydrate solution, ethylene glycol and ethanol (1:2:1 volume ratio) were used. With optimized printing parameters, no large cracks, fissures and big pores were found in the final sintered interconnects. Only dense pores with several hundreds of nanometers in diameter appeared (Figure 3h) and the square resistances were about 1.2 Ω/□.

### 3.3. Influence of the Sintering Processes

**Figure 4:**
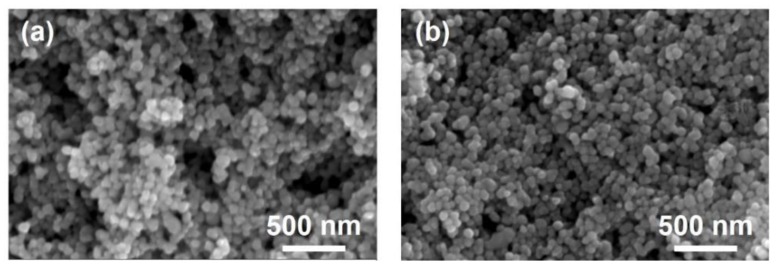
Microstructures of the interconnects sintered with (**a**) the one-step and (**b**) the two-step processes, respectively.

Since fast evaporation of the ink solvent was found to be an important reason for the formation of defects like cracks and pores, the one-step sintering process was substituted by a two-step process when the mixed solvent composed of the hydrazine hydrate solution, ethylene glycol and ethanol (1:2:1 volume ratio) were used to make the ink. The solid fraction in the ink was 40 wt %. During sintering, the temperature first rose to 150 °C slowly. The as-printed structures were partially dried during the heating process since water and ethanol were removed by volatilization. By keeping the temperature constant at 150 °C for 30 min, complete drying took place. Glycol evaporated slowly at this temperature, which was about 47 °C lower than its boiling point. Then, the temperature was further increased to 250 °C to form sintering necks between the nanoparticles. Figure 4a,b were typical microstructures of the interconnects sintered with the one-step and two-step processes, respectively. The two samples were prepared with the same printing parameters. No cracks and fissures were found for both. However, much less and smaller pores were found in the interconnects treated by the two-step sintering strategy. With the improvement in the microstructures, the square resistances of the sintered interconnects were lowered from 1.2 Ω/□ to 0.36 Ω/□.

### 3.4. Special Role Played by the Pre-Treatment of the Ink on the Interconnect Properties

**Figure 5:**
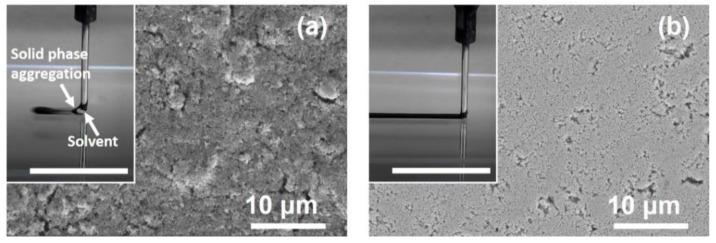
Microstructures of the interconnects printed using inks pretreated by (**a**) ultrasound and (**b**) the mechanical-ultrasonic combined method, respectively. The insets show the corresponding printing processes. The scale bars in the insets stand for 20 mm.

Both in the present and other works, the printing process frequently went wrong because a phase separation of the nanoparticle ink occurred at the nozzle exit. The phase separation could be clearly seen in the inset of Figure 5a. The ink solvent was ejected out but the solid particles blocked inside the nozzle, even when the solid fraction of the ink was as low as 20–30% and no particle aggregation was found in the ink before printing. If this happened, the copper particle blocks could be extruded out from the syringe at rather large pressure but the printed structures become discontinuous and nonuniform. To use the mixed solvent composed of the hydrazine hydrate solution, ethylene glycol and ethanol (1:2:1 volume ratio), ink was treated ultrasonically for 5 min just before being loaded in the syringe of the printer, thus alleviating this problem to some extent. However, the extrusion resistance was still uneven and the thickness of the printed structure showed great inhomogeneity. Dense cracks and voids were found in the interconnects after sintering, as shown in Figure 5a.

It was found that a pre-treatment of the ink by simultaneous ultrasonic and mechanical agitation for 5 min could solve the problem of phase separation. The mechanical agitation was made by a runner rotating at about 120 rpm. Figure 5b illustrated the interconnect microstructures obtained with the same ink, the same printing parameters and the same sintering process as those for preparing the interconnect shown in Figure 5a. Though some micro-pits were visible, the surface of the interconnect was quite smooth and the microstructure was free of cracks and large pores. 

In Fan’s work [26], copper nanoparticles were dispersed in solutions by a high shear dispersing emulsifier to fabricate stable gravure conductive ink. Joo et al. [13] also conducted a combined pretreatment of simultaneous mechanical stirring and ultrasonic processing followed by a ball milling process when dispersing micro- and nano-copper particles in a mixed solvent of diethylene glycol and PVP to prepare the conductive ink. Essentially, the high shear dispersed emulsifying process, the mechanical stirring and the ball milling all exerted shear actions on the nanoparticle dispersion.

Shearing is a common method used for preparing gel, especially in the incipient gelation stage. Structures of the gel systems are profoundly influenced by the shear rate. Lamination or network structures of different sizes emerge at comparatively low shear rates due to the enhanced migration, collision and stacking of atoms, molecules or particles. For making olive oil/monoglyceride gel, the maximum gel network developed at a moderate shear of about 300 s^−1^ [27]. At high shear rates, structure disruptions take place and the network formation is prevented.

PVP is a polymer frequently used as a film former. Crosslinking easily takes place between the long chains of the linear-type PVP molecules to produce a gel [28]. In the presence of a certain kind of polylol, for example ethylene glycol, crosslinking is promoted through chain bridging by the hydroxyls [29,30,31]. When the same amount of PVP is dissolved in water, glycol, and ethanol, the glycol solution has a viscosity 20 times higher than those of the other two [23]. This is a clear evidence to show that glycol helped to drive the gelation of PVP. 

**Figure 6:**
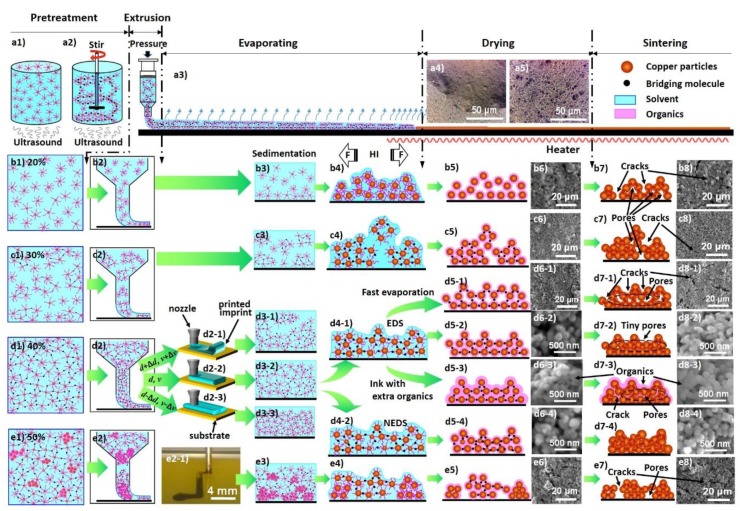
(Color online only) Schematic pictures of the printing procedures influenced by various key factors and corresponding microstructures obtained. (**a1**,**a2**) Particle configurations in the ink pretreated with and without the mechanical agitation, respectively. (**a3**) The printing procedure from extrusion to sintering. (**a4**,**a5**) Optical microscope images of the interconnect structures printed on glass substrates and dried in air for 5 min with and without the mechanical-ultrasonic combined pretreatment, respectively. (**b1**–**b8**,**c1**–**c8**,**d1**–**d8**,**e1**–**e8**) Particle configurations developed in each stage of the printing procedure using inks with the solid fractions of 20%, 30%, 40% and 50%, respectively. (**b6**–**e6**,**b8**–**e8**) Typical SEM microstructural images of the printed interconnect structures. Among them, (**d6-2**–**d6-4**,**d8-2**–**d8-4**) were obtained from the cross sections of the interconnects. The others were top views of the interconnects. (**d2-1**–**d2-3**) Schematic pictures to show influences of the printing parameters d (the distance between the nozzle and the substrate surface) and v (the moving speed of the sample platform) on the morphologies of the printed imprints when a ink with a solid fraction of 40% is applied. (**e2-1**) A macroscopic image of the printing process using a nano-copper ink with a solid fraction of 50%. Particle clusters blocked the nozzle and a discontinuous imprint was left on the substrate.

In this work, the concentration of PVP in the solution was relatively low (2 wt %). But under the conditions that the PVP chains were fully extended in the solution and glycol was presented, bridging between the PVP chains could still be effectively built to form a weak gel. The mechanical agitation exerted a shear rate of about 250 s^−1^. This might induce a laminated structure along the shear direction in the ink solution or even the onset of bridged chain network structures. The ultrasonic treatment applied simultaneously was imposed by an ultrasonic cleaner of merely 20 W. At this low energy, instead of breaking the polymer networks, the ultrasound helped to increase the collisions between particles from different laminas and led to incipient gelation. Gelation induced by the mechanical-ultrasonic combined pretreatment was illustrated schematically in Figure 6a2. Figure 6a4,a5 compare the optical microscope images of the interconnect structures printed on the glass substrate surfaces and dried in air for 5 min with and without the mechanical-ultrasonic combined pretreatment, respectively. The solid fractions in the ink for both cases were 40%. In the absence of the mechanical agitation, only local particle agglomeration due to evaporation can be found on the sample surface. While in the presence of the mechanical-ultrasonic combined pretreatment, a nicely developed hydrogel network structure throughout the whole volume was clearly seen on the top layer of the surface dried sample. At this moment, there was still a large amount of water in the network structure except the surface layer. During the subsequent drying process, the network structure would lose water slowly, collapse uniformly and gradually become dense. Such a gel structure prevented the particle aggregation as well as the phase separation and ensured the printing process to be stable.

### 3.5. Structure Inheritance in the Direct-Writing Process

Conductive ink composed of nano-metal particles is usually printed by the direct-writing process. A typical printing procedure includes: (1) pretreatment of the ink; (2) extrusion of the ink from a nozzle towards the surface of a substrate; (3) the ink spreads out on the substrate surface; (4) solvents of low boiling points in the ink evaporate slowly when the printed structure is exposed to a certain atmosphere; (5) solvents of high boiling points in the ink evaporate from the printed structure in the initial stage of sintering and the printed structures completely dry; and finally (6) sinter necks are created between nano-metal particles at high temperature and expected electrical properties are gained.

Since there are so many stages and the stages are tightly coupled with each other, it does not make much sense to optimize just one of them or even each of them. On the other hand, for different applications, various metal particles with different sizes are used. Besides, the solvents, the nozzle configurations, the substrate materials, the evaporation atmosphere, and the sintering conditions can be quite different. Optimized printing procedure in a certain case may not be good references for applications in other cases. However, to clarify the coupling mode among these stages and find out the key factors affecting the formation of the cracks and pores in each stage can be of great help.

The printing procedure, the key influencing factors and possible microstructures obtained are schematically shown in Figure 6 together with the experimental evidences. In the pretreatment stage, the solid fraction of the nanoparticles, the solvent composition in the ink and the shear rate applied for processing the ink are the key factors. According to the results in Section 3.2, the mixed solvent composed of liquid phases with different boiling points and reducing agents is a preferred choice. At a low solid fraction, nanoparticles are dispersed randomly in the liquid phase (Figure 6b1). Distances between the particles are very large and the collision probability between the particles are too low to nucleate any bridged fragments. At higher solid fraction values, the PVP chains capping around the nanoparticles start to connect with each other through the bridging molecules of glycol to form pre-gelled fragments locally with the sonication and mechanical agitation (Figure 6c1). With the solid concentration and the shear rate properly set, the particles arrange in layers in the solution and bridging among interlayer and intralayer particles takes place. An incipient gel structure or even fully developed weak hydrogel extending uniformly throughout the ink volume comes into being (Figure 6d1). When the solid content is too high, before the shear is applied, the nanoparticles partially aggregate. The aggregated clusters make the structure subsequently formed during shearing inhomogeneous (Figure 6e1).

Thin ink extruded onto the substrate surface spreads according to the ink-substrate interface energy. The solvents volatilize continuously during the evaporation and drying stages. The PVP chains curl up with the loss of solvents. The steric hindrance effect dispersing the nanoparticles diminished. Nanoparticles sediment and stack. No effective bridging creates before the particles are drawn toward each other. When the height of the residual liquid is comparable to the height of the particle stacks, the hydrodynamic interaction (HI) tears the particle stacks into patches. Even if a large number of sinter necks can form afterwards, small and dense cracks and pores already exist and the electrical properties of the obtained interconnects are rather poor (Figure 6b2–b8).

If the ink flow inside the printing nozzle during the extrusion process can be assumed to be a Newtonian fluid flowing in a laminar way within a circular tube, according to the fluid mechanics, the shear rate exerted on a certain point in the ink, can be expressed by

(1) $$\frac{dv_{n}}{dr}=\frac{\Delta P}{2l\eta }r=\frac{4\bar{v}}{R^{2}}r$$

where *v_n_* is the axial flow velocity in the nozzle, *r* the radius from the nozzle center of a certain point in the ink, Δ*P* the driving pressure of the extrusion process, *l* the length of the nozzle, *η* the dynamic viscosity of the ink, $\bar{v}$ the average flow rate inside the nozzle and *R* the inner radius of the nozzle. The shear rate varies linearly with the driving force and the average flow rate. For gelated or partially gelated ink, the flow can no longer be treated as a Newtonian one. However, in a weak gel Equation (1) may still be used as a rough estimation of the shear rate experienced by the flow inside the nozzle. The extrusion rate of the ink flow at the nozzle exit is about 0.2–1 m/s and the inner radius of the nozzle is 0.35 mm. The largest shear rate appears on the nozzle wall and is about 2300–11500 s^−1^. It is at least one order of magnitude larger than the shear rate exerted on the ink by the mechanical agitation during the pretreatment process. When the extrusion is suffering large resistances due to blocking, large driving force is to be applied and the shear rate in the nozzle can be even larger. 

To extrude ink with bridged fragments, the skeletons of the fragments within the narrow nozzle are deformed by the large shear while those out of the nozzle are still in their extended states. These fragments block each other (Figure 6c2) and relatively high extrusion pressure is necessary. At rather high extrusion pressure/shear, the bridged networks break up. The solid and liquid phases are squeezed apart and the phenomenon of phase separation appears. However, when a single piece of network is saturating the nozzle, no blocking exists and the extrusion pressure becomes lower. This leads to uneven changes in extrusion pressure. For ink with pre-gel structures mixed with aggregated clusters formed at excessive solid fraction concentrations, similar things happen (Figure 6e2). Phase separation and uneven extrusion processes give, most probably, discontinuous and rugged imprints on the substrate (Figure 6e2-1). This will be aggravated if the moving speed (v) of the sample platform is not stable or the distance (d) between the nozzle and the substrate surface is too large or too small. Even though a continuous structure is printed, the fragments stacked randomly and can be torn apart by the hydrodynamic interaction during the drying process. Consequently, large and sparse cracks and pores are left (Figure 6c7,c8,e7,e8).

At a proper solid fraction concentration (around 40%), the bridged network structure is fully developed. The network skeleton remains during extrusion and the shear exerted by the nozzle wall promotes the gelation process further. Taking into account the viscoelasticity of the weak gel structure, there is an optimal distance between the nozzle tip and the substrate surface to write a uniform imprint (Figure 6d2-1–d2-3). It is worth noting that the spreading of the extruded ink is not simply determined by the wetting of the ink solvent on the substrate surface any more. The gel network greatly influences the ink spreading process and consequently the precision of the printed structures (related results will be published elsewhere). With controlled evaporation and drying (for example, when a mixed solvent composed of constituents with various boiling points and the two-step sintering process are used), the network collapses gradually. The long organic chains remain to be bridged and connected (Figure 6d5-2–d5-4). This is illustrated under SEM by an organic layer covering the whole printed interconnect as shown in Figure 6d6-2–d6-4. The thicker the layer, the less the defects like cracks and large pores form during evaporation and drying. But this also means more residual organics. After being sintered at high temperature, this layer of organics decomposes. If a lot of organics exist and they decompose quickly, secondary pores are generated (Figure 6d7-3,d8-3). To prevent cracking and cavitation during evaporation, drying and sintering, the concentration of PVP must be carefully controlled and the two-step sintering process is preferred. Specially, the volatization rate of the ink solvents cannot be too high since the skeletons of the bridged networks collapse in homogeneously in this case to form cracks and pores (Figure 6d5-1–d8-1). 

Structures formed in the ink have great influences on the structures formed during extrusion, evaporation, drying and even after sintering. The microstructure of the final interconnect possesses a firm inheritance to the structure in the initial ink. Regulation of the final interconnect structures and properties cannot rely on optimizing certain intermediate stages, but must start from the pretreatment. If the pretreatment induces fully-developed bridged networks in the ink, compact interconnects free of cracks and large pores can be obtained. Meanwhile, gelation can help in preventing the phase separation during printing and the overspreading of the ink on the substrate. This makes printing with better uniformity and precision possible.

Another important factor influencing the compactness of the final sintered interconnects is the presence of hydrazine hydrate in the solution in this work. Hydrazine hydrate removes the oxidized outer layers of the copper particles and reduces the residual copper salts in the ink to produce some very small nano-copper particles. On the one hand, this lowers the sintering temperature and the electrical resistivity of the final interconnects. On the other hand, this leads to a compact packing of the nanoparticles according to the nonequal diameter sphere (NEDS) model instead of the equal diameter sphere (EDS) model, as shown in Figure 6d7-4 in comparison to Figure 6d7-2. Less and smaller pores are produced and the most sinter necks are to be formed. The best properties of the interconnects shall be realized through the route (a2)-(d1)-(d2)-(d2-2)-(d3-2)-(d4-2)-(d5-4) & (d6-4)-(d7-4) & (d8-4) in Figure 6.

### 3.6. Preparation and Property Tests of Crack-Free Copper Interconnects

**Figure 7:**
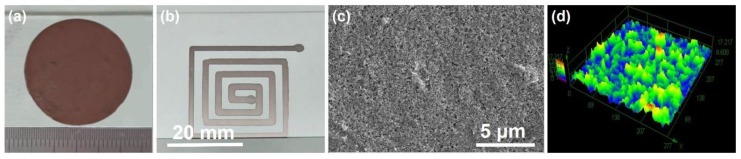
(**a**) A copper film and (**b**) an interconnect wire printed by the optimized single-run direct-writing procedure. Metallic luster of the interconnect wire could be clearly seen in (**b**) from the corresponding view angle. (**c**) A typical SEM image and (**d**) a 3D optical image of the top surface layer of the interconnect wire shown in (**b**).

Crack-free copper films and interconnect wires were successfully printed by direct-writing of a single run through the above optimized route and typically shown in Figure 7. The ink contained 40 wt % Cu nanoparticles. The mixed solvent composed of the hydrazine hydrate solution, ethylene glycol and ethanol (1:2:1 volume ratio) was applied. The pretreatment with simultaneous sonication and mechanical agitation for 5 min just before printing was conducted. The interconnect structures were fabricated at a constant printing speed (v) of 50 mm/s. The distance between the nozzle tip and the substrate d was 0.3 mm. After being sintered with the two-step strategy at a temperature of 250 °C, the films and wires had smooth surfaces (with the surface roughness smaller than 1.5 μm, Figure 7c,d) and rather good metallic luster (Figure 7b). No cracks and large pores were found under SEM. Only tiny pits with diameters smaller than 300 nm were illustrated in the microstructure images of the circuit cross sections (Figure 6d8-4 and Figure 7c). The thicknesses of the sintered films and wires could be controlled in the range of 5-50 μm (printed with nozzles of different inner diameters) and the square resistances were measured to be as low as 0.01 Ω/□. 

## 4. Conclusions

The process of printing interconnect structures by direct-writing of nano-metal particle ink on a certain substrate goes through six stages: pretreatment and extrusion of the ink, spreading, evaporation and drying of the ink trails, and sintering. A structural inheritance throughout the whole printing process was found and studied in this work. The cracks and pores detected in the final interconnects might originate from any of these stages, even the pretreatment of the ink before printing. Regulation of the final interconnect structures and properties cannot just rely on optimizing certain intermediate stages. Full consideration must be taken on the coupling effects among these stages.

By investigating the influences of the ink properties, the pre- and post-treatment processes, an optimum process route was developed for producing crack-free copper interconnects by a single-run direct-writing approach. Accordingly, the structural control started from the pretreatment of the ink before printing. With the properly controlled nanoparticle fraction, solvent properties and shear actions, weak gelation took place in the presence of long-chain polymers (e.g., PVP) and bridging molecules (e.g., glycol) before the ink was extruded out from the printing nozzle. If a complete gel network was formed, it spanned the whole volume of the solvent liquid. This not only prevented the phase separation of the ink but also helped in controlling the spreading and forming of the ink on the substrate surfaces. As long as the solvent evaporation was slow enough, the ink gel network collapsed uniformly to generate a closely packed metal particle aggregation. To achieve this, a mixed ink solvent composed of liquids with different polarities, melting points and reducibility and a two-step sintering process were applied. Compact, crack-free interconnects were successfully printed by this single-run direct-writing approach. Instead of cracks and large pore, only tiny pores smaller than 300 nm were detected in the sintered interconnects. The square resistances of the interconnects were 0.01 Ω/□ and the surface roughness was lower than 1.5 μm.

## References

[1] McAlpine M.C., Kong Y.L. (2018). 3D Printed Active Electronic Materials and Devices. U.S. Patent.

[2] Singh R., Singh E., Nalwa H.S. (2017). Inkjet printed nanomaterial based flexible radio frequency identification (RFID) tag sensors for the internet of nano things. RSC Adv..

[3] Lee J., Jun J., Na W., Oh J., Kim Y., Kim W., Jang J. (2017). Fabrication of sinter-free conductive Cu paste using sub-10 nm copper nanoparticles. J. Mater. Chem..

[4] Kaltenbrunner M., Sekitani T., Reeder J., Yokota T., Kuribara K., Tokuhara T., Drack M., Schwödiauer R., Graz I., Bauer-Gogonea S. (2013). An ultra-lightweight design for imperceptible plastic electronics. Nature.

[5] Chen Y., Au J., Kazlas P., Ritenour A., Gates H., McCreary M. (2003). Flexible active-matrix electronic ink display. Nature.

[6] Ankireddy K., Druffel T., Vunnam S., Filipič G., Dharmadasa R., Amos D.A. (2017). Seed mediated copper nanoparticle synthesis for fabricating oxidation free interdigitated electrodes using intense pulse light sintering for flexible printed chemical sensors. J. Mater. Chem..

[7] Kim S.J., Lee J., Choi Y.-H., Yeon D.-H., Byun Y. (2012). Effect of copper concentration in printable copper inks on film fabrication. Thin Solid Films.

[8] Kang J.S., Kim H.S., Ryu J., Thomas Hahn H., Jang S., Joung J.W. (2010). Inkjet printed electronics using copper nanoparticle ink. J. Mater. Sci. Mater. Electron..

[9] Kanzaki M., Kawaguchi Y., Kawasaki H. (2017). Fabrication of Conductive Copper Films on Flexible Polymer Substrates by Low-Temperature Sintering of Composite Cu Ink in Air. ACS Appl. Mater. Interfaces.

[10] Yong Y., Yonezawa T., Matsubara M., Tsukamoto H. (2015). The mechanism of alkylamine-stabilized copper fine particles towards improving the electrical conductivity of copper films at low sintering temperature. J. Mater. Chem..

[11] Konoplev B., Ryndin E., Isaeva A., Denisenko M. (2017). System of Surface Defect Monitoring Based on a Distributed Crack Sensor. Electronics.

[12] Li C., Jiang B., Wang Z., Li Y., Hossain M.S.A., Kim J.H., Takei T., Henzie J., Dag Ö., Bando Y., Yamauchi Y. (2016). First Synthesis of Continuous Mesoporous Copper Films with Uniformly Sized Pores by Electrochemical Soft Templating. Angew. Chem. Int. Ed..

[13] Joo S.-J., Hwang H.-J., Kim H.-S. (2014). Highly conductive copper nano/microparticles ink via flash light sintering for printed electronics. Nanotechnology.

[14] Lei T.G., Calata J.N., Lu G.-Q., Chen X., Luo S. (2010). Low-Temperature Sintering of Nanoscale Silver Paste for Attaching Large-Area (>100 mm^2^) Chips. IEEE Tran. Comp. Packag. Technol..

[15] Yu J.H., Kang K.-T., Hwang J.Y., Lee S.-H., Kang H. (2014). Rapid sintering of copper nano ink using a laser in air. Int. J. Precis. Eng. Manuf..

[16] Joo M., Lee B., Jeong S., Lee M. (2012). Comparative studies on thermal and laser sintering for highly conductive Cu films printable on plastic substrate. Thin Solid Films.

[17] Thiery J., Keita E., Rodts S., Murias D.C., Kodger T., Pegoraro A., Coussot P. (2016). Drying kinetics of deformable and cracking nano-porous gels. Eur. Phys. J. E.

[18] Yang K., Özçelik V.O., Garg N., Gong K., White C.E. (2018). Drying-induced atomic structural rearrangements in sodium-based calcium-alumino-silicate-hydrate gel and the mitigating effects of ZrO_2_ nanoparticles. Phys. Chem. Chem. Phys..

[19] Dugyala V.R., Lama H., Satapathy D.K., Basavaraj M.G. (2016). Role of particle shape anisotropy on crack formation in drying of colloidal suspension. Sci. Rep..

[20] Yang A.C.M., Brown H.R. (1986). Solvent Induced Crack-Like Defects in Adhered Polyimide Films. MRS Online Proc. Lib. Arch..

[21] Li Y., Tang X., Zhang Y., Li J., Lv C., Meng X., Huang Y., Hang C., Wang C. (2014). Cu nanoparticles of low polydispersity synthesized by a double-template method and their stability. Colloid Polym. Sci..

[22] Li Y., Li C., Huo Y., Lv C., Wang H. (2016). Copper Nanoparticle Ink Stabilized by Poly(vinylpyrrolidone)-Sodiumdodecylsulphate. J. Nanosci. Nanotechnol..

[23] PVP-Research-Center (2015). Rheological Properties of PVP Solution of Polyvinylpyrrolidone. http://blog.sina.com.cn/s/blog_8740acd00102w4zv.html.

[24] Jirgensons B. (1952). Solubility and fractionation of polyvinylpyrrolidone. J. Polym. Sci..

[25] Fikentscher H., Herrle K. (1945). Polyvinylpyrrolidone. Mod. Plastics.

[26] Fan X., Mo L., Li W., Li W., Ran J., Fu J., Zhao X., Li L. Synthesis of nano-copper particles for conductive ink in gravure printing. Proceedings of the 8th Annual IEEE International Conference on Nano/Micro Engineered and Molecular Systems.

[27] Ojijo N.K., Neeman I., Eger S., Shimoni E. (2004). Effects of monoglyceride content, cooling rate and shear on the rheological properties of olive oil/monoglyceride gel networks. J. Sci. Food Agric..

[28] Senogles E., Thomas R. (2007). Polymerization kinetics of N-vinyl pyrrolidone. J. Polym. Sci. Polym. Symp..

[29] Encinas M.V., Lissi E.A., Quiroz J. (1992). 2′, 2′-azobis(2-amidinopropane) as a photoinitiator of vinyl pyrrolidone polymerization in aqueous solution. Eur. Polym. J..

[30] Güven O., Yiǧit F. (1977). Effect of association on the gelation of aqueous poly (N vinyl 2 pyrrolidone) solutions with y-rays. Radiat. Phys. Chem..

[31] Darwis D., Hilmy N., Hardiningsih L., Erlinda T. (1993). Poly(N-vinylpyrrolidone) hydrogels: 1. Radiation polymerization and crosslinking of N-vinylpyrrolidone. Radiat. Phys. Chem..

